# An integrated method for cancer classification and rule extraction from microarray data

**DOI:** 10.1186/1423-0127-16-25

**Published:** 2009-02-24

**Authors:** Liang-Tsung Huang

**Affiliations:** 1 Department of Computer Science and Information Engineering, Mingdao University, Changhua 523, Taiwan

## Abstract

Different microarray techniques recently have been successfully used to investigate useful information for cancer diagnosis at the gene expression level due to their ability to measure thousands of gene expression levels in a massively parallel way. One important issue is to improve classification performance of microarray data. However, it would be ideal that influential genes and even interpretable rules can be explored at the same time to offer biological insight.

Introducing the concepts of system design in software engineering, this paper has presented an integrated and effective method (named X-AI) for accurate cancer classification and the acquisition of knowledge from DNA microarray data. This method included a feature selector to systematically extract the relative important genes so as to reduce the dimension and retain as much as possible of the class discriminatory information. Next, diagonal quadratic discriminant analysis (DQDA) was combined to classify tumors, and generalized rule induction (GRI) was integrated to establish association rules which can give an understanding of the relationships between cancer classes and related genes.

Two non-redundant datasets of acute leukemia were used to validate the proposed X-AI, showing significantly high accuracy for discriminating different classes. On the other hand, I have presented the abilities of X-AI to extract relevant genes, as well as to develop interpretable rules. Further, a web server has been established for cancer classification and it is freely available at .

## Background

The challenge of cancer treatment is to develop specific therapies based on distinct tumor types, to maximize efficacy and minimize toxicity. Hence, improvements in cancer classification have been paid more and more attention. Recently, microarray gene expression data has been successfully used to investigate useful information for cancer classification at the gene expression level. One of the earliest methods for cancer classification is the weighted voting machine which is based on a linear model [1]. Other methods includes hierarchical clustering [2], machining learning [3,4], compound covariate [5], shrunken centroids [6], partial least square [7], principal component analysis disjoint models [8], factor mixture models [9], consensus analysis of multiple classifiers using non-repetitive variables [10] etc. On the whole, these methods are mostly concentrated in the improvement of accuracy rather than other issues.

In addition to classification, another challenge is to extract relevant genes, even creditable and interpretable rules from microarray gene expression data to offer biological insight between genes. Several kinds of rules have been successfully developed in different subjects of molecular biology. In our earlier studies, decision rules based on decision tree algorithms have been effectively extracted from the thermodynamic database of proteins and mutants to explore potential knowledge of protein stability prediction [11-13]. On the other hand, association rule techniques can also reveal relevant associations between different items. Borgelt and Berthold [14] presented an algorithm to find fragments in a set of molecules that help to discriminate between different classes of activity in a drug discovery context. Oyama et al. [15] proposed a data mining method to discover association rules related to protein-protein interactions. Moreover, association rules which demonstrate diverse mutations and chemical treatments have been reported from 300 gene expression profiles of yeast [16]. Carmona-Saez et al. [17] have offered an approach which integrates gene annotations and expression data to discover intrinsic associations.

Typically, a classification system may achieve high accuracy by non-linear models, but these models are hard to provide rules. In contrast, a rule extraction system is necessary to consider the model interpretability which can provide a pathway to explore underlying relationships among data; however, this restriction often affects the system performance in classification. Hence, a learning model which can provide accurate classification, as well as useful rules, would be ideal. Even so, a relatively few attempts have been made to integrate the two types of systems on microarray gene expression data. In earlier reports, Li et al. [18] has proposed a classifier named PCL (prediction by collective likelihoods) which is based on the concept of emerging patterns and can provide the rules describing the microarray gene expression data. Tan et al. [19] have introduced a new classifier named TSP (top scoring pair) which is based on relative expression reversals and can generate accurate decision rules. These studies also revealed the phenomenon of trade-off between credibility and comprehensibility in such a hybrid system. For that reason, I have made attempts to design an integrated and effective framework with less interaction between cancer classification and rule extraction functions.

In this paper, I have presented an integrated method (named X-AI) which is based on a three-tiered architecture from the viewpoint of system design of software engineering. Different tests have been carried out on two leukemia datasets for evaluating the performance of X-AI. The obtained results indicated that X-AI is able to perform well on both functions of classification and rule extraction in microarray analysis.

## Materials and methods

### Datasets and pre-processing

I used two different leukemia datasets for the following reasons: (i) both datasets have been analyzed and discussed in many literatures, which is helpful to compare with their results; (ii) the rules extracted from the similar cancer type of datasets could be compared to each other; (iii) the robustness of classification system could be observed by the datasets that are obtained from different experiments; and (iv) the two datasets represent the nature of the binary classification and multi-class problems, which is useful to evaluate the effectiveness of the proposed method for different classification problems.

The first acute leukemia data (named L1) of Golub et al. [1] is composed of 72 samples from two different types of acute leukemia, acute lymphoblastic leukemia (ALL) and acute myeloid leukemia (AML). The training set has 38 bone marrow samples (27 ALL and 11 AML) and the test set consists of 24 bone marrow and 10 peripheral blood samples (20 ALL and 14 AML). Bone marrow mononuclear cells were collected by Ficoll sedimentation in the training set and RNA was hybridized to Affymetrix oligonucleotide microarrays, by which each sample has expression patterns of 7129 probes measured. The second acute leukemia data (named L2) of Armstrong et al. [20] includes 12582 gene expression values for 57 peripheral blood or bone marrow samples. The training set contains 57 leukemia samples (20 ALL, 17 MLL (mixed lineage leukemia) and 20 AML) and the test set contains 15 samples (4 ALL, 3 MLL and 8 AML). For microarray data, pre-processing is of critical importance in downstream analyses. In order to equalize expression values for each sample and avoid the bias against samples, all values in a sample have been re-scaled by a multiplicative factor which is determined by linear regression of genes with present calls. All multiplicative factors are available on the established web server. Duoit et al. [21] applied thresholding, filtering and logarithmic transformation steps before analyzing the leukemia dataset. Accordingly, the expression values were limited by both upper and lower bounds. Since it could be easy to neglect information leakage effects during pre-processing of the proteomic profiling on mass spectrometry data as well as the microarray expression data [22], the upper bound is lifted to 24000 and the lower bound -800, which can increase the changes of finding relevant genes due to a larger search space. Further, I tried to perform the feature selection function instead of a simple filter to systematically reduce the number of genes. The mechanism is described in the following section.

More details of datasets can be found on the web server and in Broad Institute  which evolved from research collaborations in the MIT and Harvard communities and made the generated data available to the scientific community.

### X-AI Method

**Figure 1:**
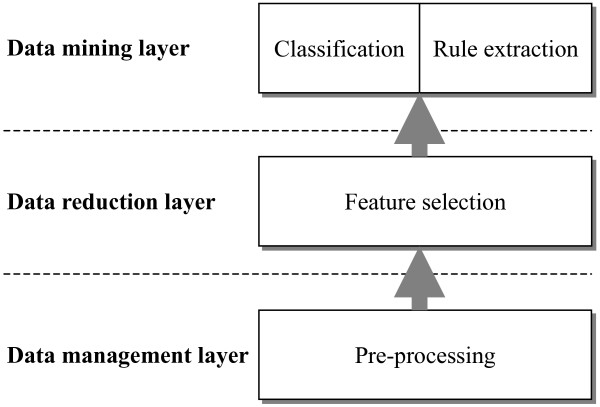
**A three-tiered architecture applied to microarray gene expression data to integrate the tasks of data analysis from the pre-processing to the data mining**.

From the viewpoint of system design in software engineering, Yourdon and Constantine [23] made a major contribution to the development of structured design methods by defining a series of criteria that can be used in separating systems into appropriate modules. Modules with tight cohesion and loose coupling are the goal of design. Tight cohesion means that a module should capture one abstraction, while loose coupling means that modules should have little dependency on each other. Introducing the concepts, I adopted a three-tiered architecture (see Figure 1) for the integrated system and each layer includes one or more specific functions: (i) The data management layer comprises the functions required at all stages of data pre-processing issues in microarray analysis. This is consistent with the report of Tinker et al. [24], describing the data management is necessary for the pre-processing which is an important part of microarray experimentation. (ii) The data reduction layer corresponds to the feature selection function, which is mainly to reflect the fact that not all genes measured from a microarray are relevant to a particular cancer; moreover, the data reduction can also help to reduce computational complexity. (iii) The data mining layer satisfies the functions of different kinds of analysis, and here is partitioned into two functions of classification and rule extraction. The two functions based on the same lower layer are loosely coupled and each delivers a coherent group of services, conforming to the design principle mentioned above.

The three-tiered architecture integrates the tasks of microarray data analysis from the pre-processing to the data mining including classification and rule extraction. Each function layer with independency can be changed internally without affecting other layers. Therefore, this architecture can provide the consistency of data to different components of the same layer, and reduce the interaction between layers as well as between the components of the same layer.

**Figure 2:**
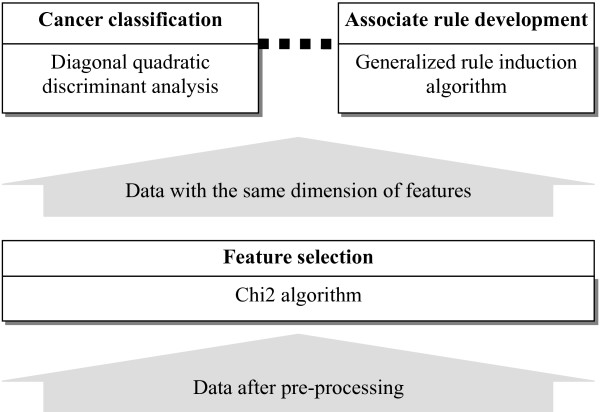
**The X-AI framework with dataflow for cancer classification and knowledge acquisition from DNA microarray data**.

The proposed X-AI method primarily implemented the data mining and the data reduction layers of the architecture, and integrated three functions: (i) feature selection, (ii) cancer classification, and (iii) associate rule development (see Figure 2). Although there are many algorithms for these functions, I included three common algorithms so as to observe how well the integrated architecture can perform. Nevertheless, it is optional that replacing these algorithms with others which conform to these functions. Here, Chi2 algorithm serves as the selector to systematically extract the relative important genes so as to reduce the dimension and retain as much as possible of the class discriminatory information. This selector can also provide the consistency of data to the other functions, the input data flows of which come from the output data flows of the selector. Subsequently, diagonal quadratic discriminant analysis (DQDA) was combined to discriminate tumor classes. And generalized rule induction (GRI) was integrated to establish association rules which can give an understanding of the relationship between cancer classes and influence genes. In addition, the outcomes obtained from the three functions of selector, classification and rule development can be referenced by each other. For example, an accurate classification reveals the fact that the selected features are effective, which generally makes the developed rules more reliable.

#### Chi2 algorithm

The Chi2 algorithm [25] can discretize numeric features and select relevant features according to the chi-squared statistic with respect to the class. The chi-squared value of an attribute is calculated as the following equation,

(1) $$\chi^{2}=\sum_{i=1}^{2}\sum_{j=1}^{\text{k}}\frac{{\left(A_{ij}-E_{ij}\right)}^{2}}{E_{ij}},$$

where k is the number of classes and *A*_*ij *_the number of samples of the *j*-th class in the *i*-th interval. *E*_*ij *_means the expected frequency of *A*_*ij*_, which is calculated by

(2) $$E_{ij}=\frac{R_{i}*C_{j}}{n},$$

where *R*_*i *_is the number of samples in the *i*-th interval, *C*_*j *_the number of samples in the *j*-th class, *n *the total number of samples. The algorithm mainly consists of two phases, named Phase I and II. Phase I comprises the calculation of the chi-squared value for adjacent intervals, and the merge of adjacent intervals under a chi-squared threshold which will be decrementing until an inconsistency rate of data is exceeded; Phase II includes the finer process of Phase I for each feature, and the evaluation of the merge degree which reveals the relevant feature to data. For example, a feature is regarded as an irrelevance for data if it is merged to only one value at the end of Phase II.

In this work, I have applied the algorithm to two different datasets to analyze the relative importance of genes for the discrimination of tumor classes. And it was chiefly carried out from a suit of free open-source software [26], which provides numerous machine learning algorithms from various learning paradigms.

#### Diaquadratic discriminant analysis (DQDA)

Based on Bayes decision theory, the maximum likelihood (ML) discriminant rule discriminates the class of a feature vector *x *by assigning the one which yields maximal likelihood [27]. For multivariate Gaussian distributions, the likelihood function of *ω*_*i *_with respect to *x *in the *l*-dimensional feature space is given by

(3) $$p(x|\omega_{i}\text{)=}\frac{1}{{\left(2\pi \right)}^{l/2}|\Sigma_{i}{|}^{1/2}}\exp[-\frac{1}{2}{\left(x-\mu_{i}\right)}^{T}\Sigma_{i}^{-1}(x-\mu_{i})],$$

where *μ*_*i *_is the mean of *x *for the *ω*_*i *_class, Σ_*i *_the *l *by *l *covariance matrix. When the covariance matrices are diagonal, $\Sigma_{i}=\text{diag}(\sigma_{i\text{1}}^{\text{2}},...,\sigma_{il}^{\text{2}})$, the ML discriminate rule can be written as $C(x)=\underset{i}{\arg\min}\sum_{j=1}^{l}\left[{\left(x_{j}-\mu_{ij}\right)}^{2}/\sigma_{ij}^{2}+\log\sigma_{ij}^{2}\right]$, which is a special case of diagonal quadratic discriminant analysis (DQDA). In practice, *μ*_*i *_and Σ_*i *_are estimated by corresponding sample quantities. we have effectively utilized it for the analysis of discriminating two- and three-state proteins [28]. In this study, the combination of selected genes was used as the feature vector to discriminate tumor classes.

#### Generalized rule induction (GRI)

Generalized rule induction was proposed by Smyth and Goodman [29], which applies an information theoretic approach to automate rule acquisition. For a rule, *if antecedent then consequent*, GRI applies *J*-measure quantifies its information content:

(4) $$J=p(a)\left[p(c|a)\ln\frac{p(c|a)}{p(c)}+[1-p(c|a)]\ln\frac{1-p(c|a)}{1-p(c)}\right],$$

where *p*(*a*) represents the probability of the observed attribute value of *a*, as a measure of the coverage of the antecedent; *p*(*c*) represents the prior probability of the value of *c*, as a measure of the common of the observed attribute value of c in the consequent; *p*(*c*|*a*) represents an modified probability of observing this value of *c *after taking into account the additional information of the value of *a*. For rules with more than one antecedent, *p*(*a*) is regarded as the probability of the conjunction of the variable values in the antecedent. Accordingly, a set of optimal rules was then generated by ITRULE algorithm, which calculates *J*-measures of rules by employing depth-first search over possible left-hand sides.

Here, the genes selected by Chi2 algorithm were considered as the attributes of the antecedent. And the tumor class was the only attribute of the consequent.

### Performance evaluation and test procedure

#### Prediction accuracy

I considered the classification of the leukemia datasets L1 and L2 as the two-class and three-class problems, respectively. To evaluate the performance of the classification problems, both classification accuracy and misclassified number were calculated along with corresponding number of selected genes.

#### Support and confidence

The support and confidence measures were defined to reveal the importance of individual association rule. For a particular association rule, support is the proportion of samples in the dataset that contain the rule antecedent:

(5) $$support=\frac{\text{number of samples containing antecedent}}{\text{total number of samples}}.$$

This measure reveals the comprehensiveness of the rule to the dataset.

Further, confidence of the association rule is a measure of accuracy of the rule:

(6) $$confidence=\frac{\text{number of samples containing both antecedent and concequent}}{\text{number of samples containing antecedent}}.$$

#### Holdout validation and leave-one-out cross-validation tests

The present method was validated by both holdout validation and leave-one-out cross-validation (LOOCV) tests. Holdout validation derives a predictor from the training set, and uses the blind or independent test set to evaluate the predictor. LOOCV is simple *n*-fold cross-validation, where *n *is the number of samples in the dataset. Each sample is left out in turn, and the predictor is trained on all the remaining ones. The procedure is repeated for *n *times to obtain a mean score.

## Results and discussions

### Analysis of important genes

X-AI provides a feature selection function to systematically extract the relative important genes for discriminating different classes. In Table 1, the top ten genes for each training set of two datasets are listed according to the order of the chi-squared statistic. The selected genes provide input information to both subsequent functions of classification and rule development, and the small number of selected genes has a low data dimension, as well as low calculation complexity. Nevertheless, the decision of the nmber is flexible and largely depends on the analysis requirement.

In the part of L1, the importance of most genes has been discussed in the study of Golub et al. [1] and in earlier literatures. Further, Wang et al. [30] also presented additional arguments about Zyxin and PTX3, suggesting that the expression level of both plays an important or neglected role in distinguishing between ALL and AML. The selection function of X-AI has also been compared with some other selection algorithms, including information gain and symmetrical uncertainty criteria. It showed an almost the same selection in the top ten genes. In the part of L2, the average of chi-squared values is higher than that in L1. The results indicate that most of genes extracted by the selection function of X-AI agrre with earlier studies, and may be important for the class discrimination.

**Table 1:** Top ten genes selected by feature selection function of X-AI for two datasets

Dataset	Probe ID	Gene annotation	χ^2 ^Score
L1	X95735	Zyxin	38.00
	M55150	FAH Fumarylacetoacetate	33.54
	M27891	CST3 Cystatin C (amyloid angiopathy and cerebral hemorrhage)	33.31
	M31166	PTX3 Pentaxin-related gene, rapidly induced by IL-1 beta	33.31
	X70297	CHRNA7 Cholinergic receptor, nicotinic, alpha polypeptide 7	29.77
	U46499	GLUTATHIONE S-TRANSFERASE, MICROSOMAL	29.77
	L09209_s	APLP2 Amyloid beta (A4) precursor-like protein 2	29.77
	M77142	NUCLEOLYSIN TIA-1	29.77
	J03930	ALKALINE PHOSPHATASE, INTESTINAL PRECURSOR	29.02
	M23197	CD33 CD33 antigen (differentiation antigen)	28.95
L2	36239_at	H. sapiens mRNA for oct-binding factor	91.08
	37539_at	Homo sapiens mRNA for KIAA0959 protein, partial cds	84.51
	35260_at	Homo sapiens mRNA for KIAA0867 protein, complete cds	83.72
	32847_at	Homo sapiens myosin light chain kinase (MLCK) mRNA, complete cds	79.82
	35164_at	Homo sapiens transmembrane protein (WFS1) mRNA, complete cds	79.46
	1325_at	Homo sapiens TWIK-related acid-sensitive K+ channel (TASK) mRNA, complete cds	78.57
	40191_s_at	wg66h09.x1 Homo sapiens cDNA, 3' end	77.22
	39318_at	H. sapiens mRNA for Tcell leukemia	76.22
	32579_at	Human transcriptional activator (BRG1) mRNA, complete cds	74.97
	41715_at	H. sapiens mRNA for phosphoinositide 3-kinase	73.53

L1: the dataset of Golub et al. [1]

L2: the dataset of Armstrong et al. [20]

### Prediction performance of system

**Figure 3:**
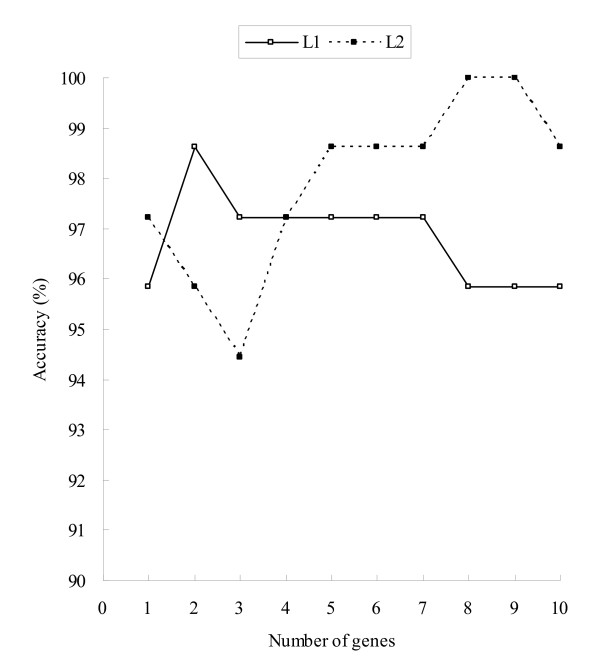
**Prediction performance of X-AI along with different number of genes on the test set of two datasets**. The y-axis represents classification accuracy and the x-axis is the corresponding number of genes which were used as information in classification. L1: for the dataset of Golub et al. [1] L2: for the dataset of Armstrong et al. [20]

Different tests have been applied to verify the accuracy of the classification function of X-AI. For holdout validation test, it shows the accuracy of 96% and 99% on the test sets of L1 and L2, respectively, using the ten genes as input information. I have also carried out the analysis of classification accuracy along with the corresponding number of genes by holdout validation test. Figure 3 illustrates the classification accuracy as a function of the number of selected genes. The genes were one by one included as the input information according to the order of chi-squared statistic. On the test set of dataset L1, X-AI achieves an accuracy of 98.6% using two genes, and increasing the number of genes to 10 did not further improve it. In addition, on the test set of dataset L2, the accuracy can increase to 100% using eight genes. On the one hand, the training and test sets for each dataset were combined to form a complete dataset for LOOCV test. The test yielded the accuracy of 96% and 94% for datasets L1 and L2, respectively.

The results show that the classification function performs well in discriminating these different classes when the input information is provided by the feature selector function of X-AI. Namely, the integration of the both functions can be feasible and effective for the binary classification and three-class problems.

### Comparison with other methods

**Figure 4:**
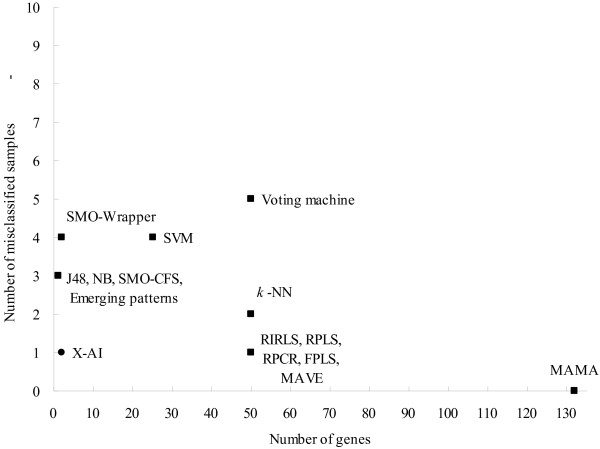
**Comparison of prediction performance between different methods**. The y-axis denotes the number of samples which were misclassified by those methods on the test set of L1. The number of used genes is represented in the x-axis. Voting machine [1] SVM [31] Emerging patterns [32] MAMA [33] J48, NB, SMO-CFS, SMO-Wrapper [30] RIRLS, RPLS, RPCR, FPLS, MAVE, *k*-NN [34]

The performance comparison between X-AI and other methods has also been made on different datasets. The results provide an overall view about the performance of different methods. In Figure 4, the prediction performance is tested on dataset L1 by holdout validation. These compared methods include the weighted voting machine, which is based on a linear model [1]; support vector machines (SVM) [31]; the emerging patterns algorithm [32]; maximal margin linear programming (MAMA) [33]; four methods that combine the feature selector with machine learning algorithms [30] and six methods which have been discussed in earlier literature [34]. The numbers of misclassified samples and of used genes vary from 0 to 5 and 1 to 132, respectively. This analysis shows that other methods can not dominate X-AI simultaneously on the numbers of misclassified samples and of used genes; namely, X-AI has a relatively small number of misclassified samples or used genes.

**Figure 5:**
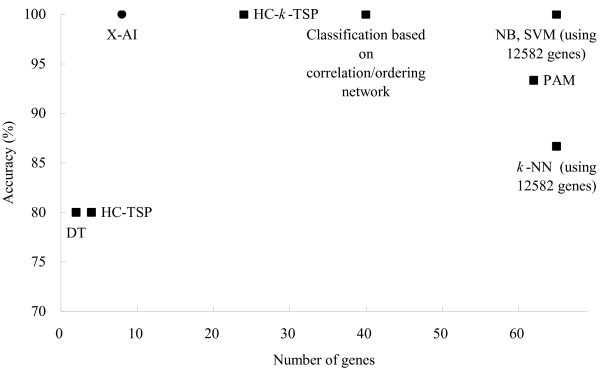
**Comparison of prediction performance between different methods**. The y-axis denotes the number of samples which were misclassified by those methods on the test set L2. The number of used genes is represented in the x-axis. Classification based on correlation/ordering network [35] HC-TSP, HC-*k*-TSP, DT, NB, *k*-NN, SVM, PAM [19]

Figure 5 shows the comparison of prediction performance on dataset L2. the classification based on correlation/ordering network [35] showed an accuracy of 100% using information of 40 genes. Other seven compared methods include three TSP-family classifiers and five machine learning methods: C4.5 decision trees (DT), Naïve Bayes (NB), *k*-nearest neighbor (*k*-NN), SVM and prediction analysis of microarrays (PAM) [19]. The accuracy and the number of used genes vary from 80% to 100% and 2 to 12582, respectively. The analysis reveals that X-AI can achieve a relatively high accuracy using a small number of informative genes when comparing to these methods.

### Association rule development

The function of feature selection did not only reduce the number of input genes, but also improve the efficiency of rule development. It also results in a rational and acceptable number of rules. Based on the genes of Table 1, X-AI included all the samples for each dataset to establish association rules.

Tables 2 and 3 list all association rules that developed for each dataset and class. The average confidence is 99% and 97% for datasets L1 and L2, respectively, showing the high accuracy of these rules. In Table 2, the second rule means that *if the expression of M23197 (CD33) is larger than 401.5*, *then the sample is classified as ALL*. For dataset L1, 29.17% samples contain the antecedent of this rule and all these samples are correctly classified. This rule efficiently reveals the importance of the gene in discriminating between AML and ALL. This finding is in accord with the results of earlier studies [1,19]. Further, I observed the occurrence of genes among the rules, which may related to their importance. Interestingly, the gene X95735 (Zyxin) has a highest percentage of occurrence (30%) and Wang et al. [30] also gave a detailed discussion about its role in leukemia. In Table 3, the gene 1325_at (TASK) also has a high percentage of occurrence (24%). However, it may need more comparative studies for validation.

**Table 2:** Two different classes of rules generated from dataset L1

Consequent	Antecedent	Support (%)	Confidence (%)
ALL	L09209_s > 1056.5 & M23197 > 326.0	30.56	100
	M23197 > 401.5	29.17	100
	M27891 > 2096.5	27.78	100
	X95735 > 994.0 & M55150 > 1250.5	27.78	100
	X95735 > 994.0	36.11	92
AML	U46499 < 154.5	59.72	100
	L09209_s < 992.5	58.33	100
	X95735 < 994.0	63.89	98
Mean		41.67	99

**Table 3:** Three different classes of rules generated from dataset L2

Consequent	Antecedent	Support (%)	Confidence (%)
ALL	32847_at > 147.0	30.56	100
	36239_at > 2201.0	27.78	100
AML	39318_at < 1063.0 & 32579_at < 2285.0	34.72	100
	1325_at < 1501.5, 39318_at < 1063.0 & 32579_at < 2285.0	34.72	100
	1325_at < 1501.5, 36239_at < 214.0 & 40191_s_at < 508.5	33.33	100
	36239_at < 214.0 & 40191_s_at < 508.5	33.33	100
	39318_at < 1063.0 & 35164_at < -794.5	31.94	100
	40191_s_at < 519.0 & 36239_at < 167.0	31.94	100
	1325_at < 1501.5, 39318_at < 1063.0 & 35164_at < -794.5	31.94	100
	1325_at < 1501.5, 40191_s_at < 519.0 & 36239_at < 167.0	31.94	100
	1325_at < 1501.5, 36239_at < 214.0 & 37539_at < -362.0	31.94	100
	36239_at < 214.0 & 37539_at < -362.0	31.94	100
	37539_at < -725.5	29.17	100
	32579_at < 2285.0	36.11	96
	1325_at < 1501.5 & 32579_at < 2285.0	36.11	96
	36239_at < 214.0	40.28	93
MLL	1325_at < 201.0, 35260_at > 794.5 & 40191_s_at > 1107.5	19.44	100
	1325_at < 201.0 & 36239_at > 214.0	23.61	94
	1325_at < 201.0	37.50	67
Mean		32.02	97

### Web server for cancer classification

**Figure 6:**
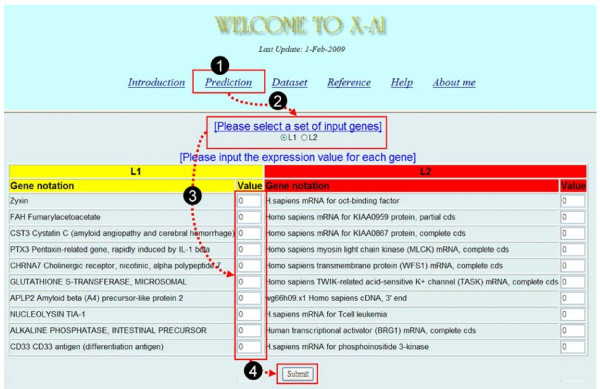
**Snapshot of the prediction page of web service for cancer classification**.

I have also developed a web server for classifying tumors of acute leukemia and it is freely available at . The prediction can be made by taking four simple steps (see Figure 6): (i) select "Prediction" from the main page to open an input subpage, (ii) select a set of input genes, (iii) input the expression values for each gene, and (iv) press the "Submit" button to start the service.

Because X-AI selected two different sets of input genes from two datasets for training the classifiers, it results in two classifiers with different sets of input genes. Users can optionally assign one of both to predict cancer classes. In addition to the cancer classification page, the web server has provided help and reference pages for interested researchers.

## Conclusion

In this study, I have proposed an integrated method for accurate cancer classification, relevant gene selection, and the associate rule development from DNA microarray data. Applying the concepts of system design, the modules in the present architecture are tight cohesion and loose coupling.

Through different tests, the method shows high classification accuracy on two leukemia datasets. In addition, the selected genes and the generated rules are in accord with recent studies. The results suggest that the method can effectively integrate these related functions for the analysis of microarray data.

## Competing interests

The author declares that they have no competing interests.
